# Non-classical Mullins-Sekerka instability during mineralization at gas-liquid interfaces

**DOI:** 10.1038/s41598-026-76568-9

**Published:** 2026-10-26

**Authors:** Lara Wegner, Ryan Santoso, Alireza Chogani, Joe Jennings, Dirk Bosbach, Jenna Poonoosamy

**Affiliations:** 1 Institute of Fusion Energy and Nuclear Waste Management (IFN-2), Forschungszentrum Jülich GmbH, Wilhelm-Johnen-Strasse, Jülich, 52425 Germany; 2 Faculty of Geosciences, Utrecht University, Princetonlaan 8a, CB Utrecht, 3584 Netherlands; 3 School of Earth & Environmental Sciences, University of St Andrews, Queen’s Terrace, St Andrews, KY16 9TS UK

**Keywords:** FIB-SEM, Mullins-Sekerka, Gas-liquid interface, Effective diffusivity, Materials science, Solid Earth sciences

## Abstract

Diffusion-driven morphological instability, described by Mullins–Sekerka theory, predicts amplification of interfacial perturbations and wavelength selection for idealized sharp interfaces. However, natural systems rarely satisfy these assumptions. Here, we investigate barite mineralization on the growing interface of CO$_{2}$ gas bubbles using 3D focused ion beam scanning electron microscopy (FIB-SEM) tomography, quantitative surface analysis and reactive transport modelling. Bubble morphologies exhibit diffusion-driven instability, with dominant modes consistent with Mullins–Sekerka predictions. However, the interface violates key assumptions of the classical framework; it is porous, transport active, and evolves in pore space confinement, while crystallization proceeds via heterogenous nucleation, producing a polycrystalline interface. We show that diffusion-driven instability persists under these conditions but with modified mode selection, defining a non-classical Mullins–Sekerka regime for gas-liquid interface mineralization. Such dynamics influence pore structure evolution and transport pathways in the subsurface environment.

## Introduction

Coupled dissolution and precipitation with gas generation occurs in geological and engineered systems during, e.g., hydrogen generation from serpentinization of ultramafic rocks^1–4^, anoxic corrosion of metallic waste canisters in deep geological repositories for radioactive waste^5,6^, hydrothermal alteration, and various mineral fluid interactions in porous media. In these systems, gas exsolution introduces a moving and deformable interface on which mineralization occurs^7^. Mineral growth at this interface alters pore structure, transport properties, mineral reactivity thereby controlling gas migration^8,9^. Understanding mineral growth at gas-liquid interfaces is therefore essential for predicting and optimizing gas production (e.g., hydrogen) from mineral–fluid reactions.

Gas-liquid interfaces undergoing mineralization are non-equilibrium systems in which transport, interface geometry, and interfacial thermodynamics are coupled. When growth is diffusion-limited, flux focusing causes protruding regions of the interface to receive a higher flux, leading to amplification of small perturbations. This mechanism drives morphological instabilities and explains the emergence of interfacial structures such as undulations and branching^10^. The classical description of diffusion-driven interfacial instability is given by the Mullins-Sekerka theory, which analyzes the linear stability of a growing interface under diffusive transport and capillary effects^11^. In this framework, long-wavelength perturbations grow due to diffusion-driven flux focusing, leading to the development of interfacial undulations, whereas short-wavelength perturbations are suppressed by surface tension through the Gibbs–Thomson effect. As a result, the system selects a characteristic wavelength associated with the fastest-growing perturbations. The theory predicts the growth or decay of infinitesimal perturbations and their dependence on system parameters, but does not determine the final morphology. The resulting evolution depends on boundary conditions, geometry, and the physical constraints imposed on the growth process^12–18^.

Recently, experimental studies have shown that mineralization on the surface of growing gas bubbles produces cauliflower like morphologies, i.e., a mineral shell forming around a gas bubble with an undulating surface^7^. In these experiments, witherite (BaCO$_{3}$) dissolved under acidic conditions, producing CO$_{2}$ gas bubbles and Ba$^{2+}$, which subsequently reacted with injected sulfate ions to form barite. Mineral precipitation occurs preferentially at the gas-liquid interface, consistent with reduced nucleation barriers at the bubble surface. While the chemical pathway leading to barite formation is well understood, the physical mechanism responsible for the emergence of surface undulations remains unclear.

A likely candidate is the Mullins-Sekerka instability. However, mineral precipitation at gas–liquid interfaces violates key assumptions of the classical framework. In particular, barite growth is anisotropic, e.g., with crystallographic faces {001} growing faster than {010}, and newly formed precipitates can be porous rather than acting as a sharp, impermeable boundary^4,19^. Furthermore, in the aformentioned experiments, gas bubbles nucleate and grow within confined pore spaces, where interactions with solid boundaries further constrain interface evolution. These features introduce spatially heterogenous transport and locally variable supersaturation at the growth front, such that the effective interface differs from the smooth boundary assumed in classical analyses. As a result, both anisotropic growth, pore confinement effect, and transport through the evolving porous shell can modify the amplification and selection of interfacial modes.

This raises the question of whether the observed surface undulations arise from a classical Mullins-Sekerka-type (diffusion-driven) instability, or from a modified instability shaped by pore space confinement, porous-shell transport, and crystallization kinetics.

In this study, we investigate barite precipitation at the surface of growing CO$_{2}$ bubbles using theoretical analysis of Mullins-Sekerka instability, quantitative surface characterization, focused ion beam scanning electron microscopy (FIB-SEM) tomography, and reactive transport simulations. We show that bubble morphologies exhibit signatures of diffusion-driven instability. Dominant modes extracted from 2D SEM images morphological consistent with Mullins-Sekerka-type predictions indicating that early-stage growth is governed by diffusion-driven amplification of interfacial perturbations. However, some of the observed structures deviate from the classical Mullins-Sekerka framework. The mineral shell is porous and transport-active, leading to heterogenous supersaturation, while its polycrystalline texture and nucleation rate analysis indicate that growth is dominated by 2D heterogenous nucleation at the interface. This introduces roughening of the surface^20^. In addition, confinement within the pore space perturbs both the interface geometry and the surrounding diffusive field. These effects modify the instability beyond the classical description. While the evolution of the interface remains diffusion-driven in the Mullins-Sekerka sense, porous-shell transport, evolving diffusivity, confinement, and crystallization kinetics alter mode selection and growth dynamics, defining a non-classical Mullins-Sekerka regime for bubble-surface mineralization.

## Results

### 3D FIB-SEM characterisation and evaluation of effective diffusivity

A flow-through micro-reactor experiment with packed witherite grains (20–63 µm grain size) was conducted to investigate the coupled mineral dissolution and precipitation with gas generation, and consequential changes on transport pathways^8^. A 300 mM Na$_{2}$SO$_{4}$ solution at pH 1 was injected, inducing witherite dissolution and CO$_{2 (aq)}$ exsolution and consequent reaction of the resulting sulfate ions with the aqueous Ba$^{2+}$ to form barite. CO$_{2 (aq)}$ re-equilibrates with the aqueous pore water and is exsolved forming gas bubbles according to the following equation:

1 $$\begin{aligned} BaCO_{3 (s)} + 2H^{+} _{(aq)} + SO_{4 (aq)}^{2-} \rightleftharpoons BaSO_{4 (s)} + H_{2}O_{(l)} + CO_{2 (aq)} \end{aligned}$$

**Fig. 1:**
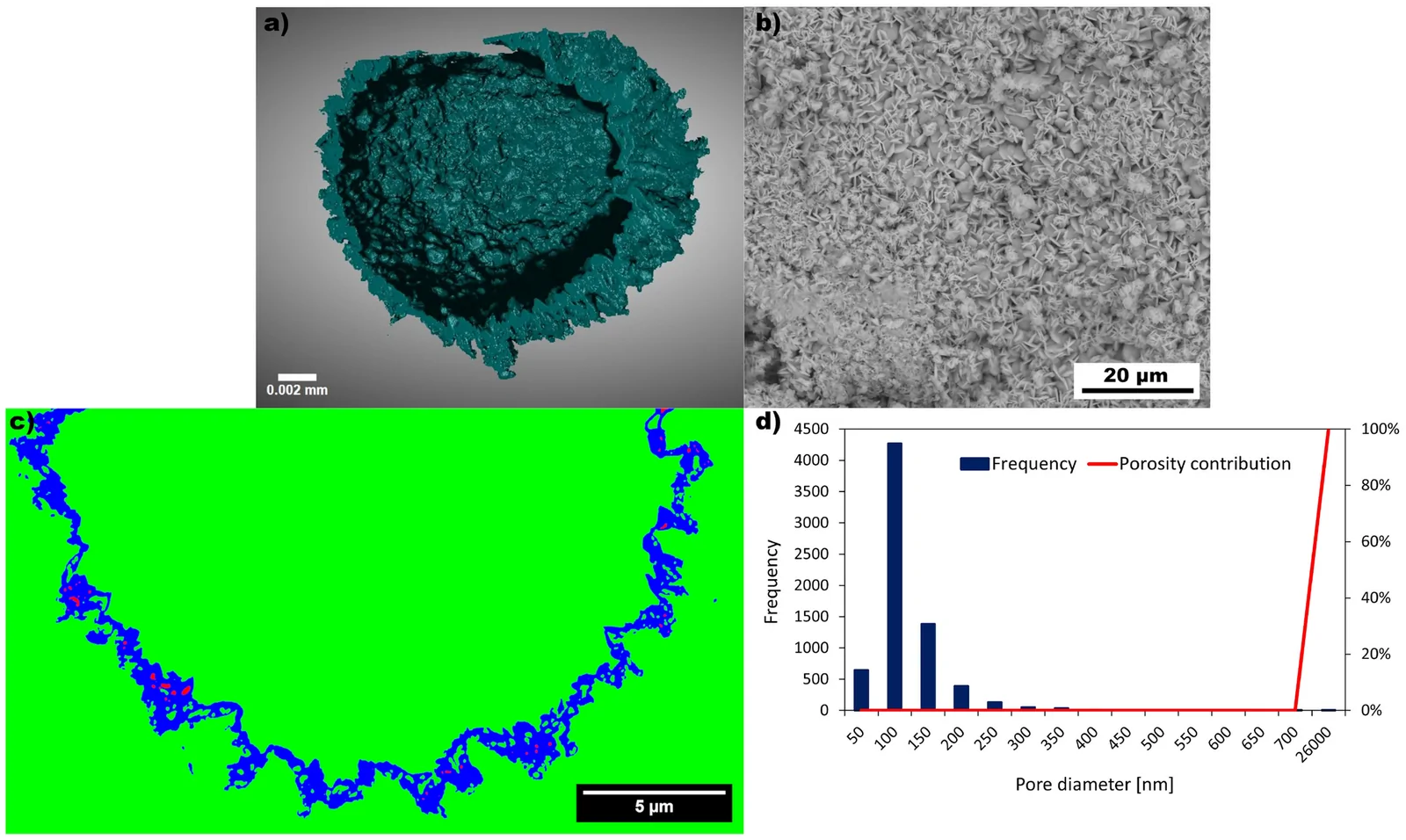
3D FIB-SEM characterization of mineral shell around the bubble. (**a**) 3D morphology of isolated gas bubble reconstructed from FIB-SEM images. (**b**) Bubble shell consisting of polycrystalline barite composed of small platelet-like crystals. (**c**) The representative slice of isolated gas bubble shows pore connectivity. Blue = barite precipitates, green = connected porosity, red = isolated porosity. (**d**) Calculated pore diameter [nm] vs frequency of pore diameter of isolated gas bubble. Please note the discontinuous scale of the x-axis. The porosity contribution is dominated by connected components.

In this study, a sample of the reacted witherite region from^8^ is analyzed using advanced electron microscopy to investigate barite mineralization on the surface of isolated CO$_{2}$ gas bubbles in the pore space. The 3D morphology reconstructed from the FIB-SEM images is shown in Fig. 1a with a magnified view of the bubble shell in 1b revealing polycrystalline barite composed of small platelet-like crystals.

Figure 1c shows one exemplary slice of the FIB-SEM images to visualize pore connectivity of the secondary mineral phase (blue). This 2D slice reveals the wavy interface of the barite shell. 3D analysis allows visualization of connected (green) and isolated components (red) in the scans. It proves that the majority of the pore volume is connected, enabling diffusion of solutes along the waterfilms of the shell^21^. Isolated pores only contribute 0.1 % to the whole pore volume. These pores do not contribute to the transport properties of the system as they cannot be accessed by fluid or gas flow. Refer to Supplementary Note S3 and Supplementary Table S1 detailing the porosity analysis, and results at other locations.

Figure 1d shows the pore size distribution and the corresponding contribution of each pore class to the total porosity. Pore sizes are on the nanometre-scale ranging from 50 to 700 nm. The distribution is dominated by smaller pores, between 50–150 nm, but their contribution to the porosity is negligible. In contrast, a small number of larger connected pores (above 700 nm) contributes significantly to the overall porosity (99.9 %). Supplementary Figure S2 provides the pore size distributions of other sampled locations and similar trends.

We examined ten random ROIs on the 3D FIB-SEM structure to evaluate effective diffusivity ($D_{e}$) of the secondary mineral precipitates. This step helps assess transport behaviour mechanism through the barite shell. Supplementary Figure S3 shows the location of ROIs on the 3D FIB-SEM scan, and for additional locations. The effective diffusivity was evaluated with Lattice Boltzmann simulation (see Section "Evaluation of effective diffusivity") on each ROI using 10 cores in JURECA High-Performance Computing Infrastructure^22^ for 2 hours. $D_{e}$ of secondary mineral precipitates yield 5.85 ± 0.95 $\times 10^{-10}$ m$^{2}$ s$^{-1}$. Effective diffusivity at other locations of secondary mineral precipitates are in the same order of magnitude or smaller $10^{-11}$ m$^{2}$ s$^{-1}$. Refer to Supplementary Table S2 for details of sizes of ROIs and individual values of $D_{e}$ and additionally Supplementary Note S4 for results of effective diffusivity at other locations.

### Consequences of evolving diffusivity on the saturation index at the gas-liquid interface

As discussed previously, the barite precipitates exhibit variation in effective diffusivity, from $10^{-10}$ to $10^{-11}$ m$^{2}$ s$^{-1}$. This variation likely reflects the temporal variation in diffusivity. In this section, we evaluate how changes in effective diffusivity affect the transport of ions and, consequently, the development of the fluid saturation index with respect to barite. The saturation index is defined as:

2 $$\begin{aligned} SI = \log _{10}\left( \frac{IAP}{K_{sp}}\right) \end{aligned}$$

where *IAP* [-] is the ion activity product, here of Ba$^{2+}$ and SO$_{4}^{2-}$ in HCl at pH 1, and $K_{sp}$ [-] is the solubility product of the corresponding mineral, here $K_{sp}$(BaSO$_{4}$) = 10$^{-9.96}$^23^. $\textrm{SI}> 1$ indicates barite precipitation, whereas $\textrm{SI} < 1$ indicates dissolution.

**Fig. 2:**
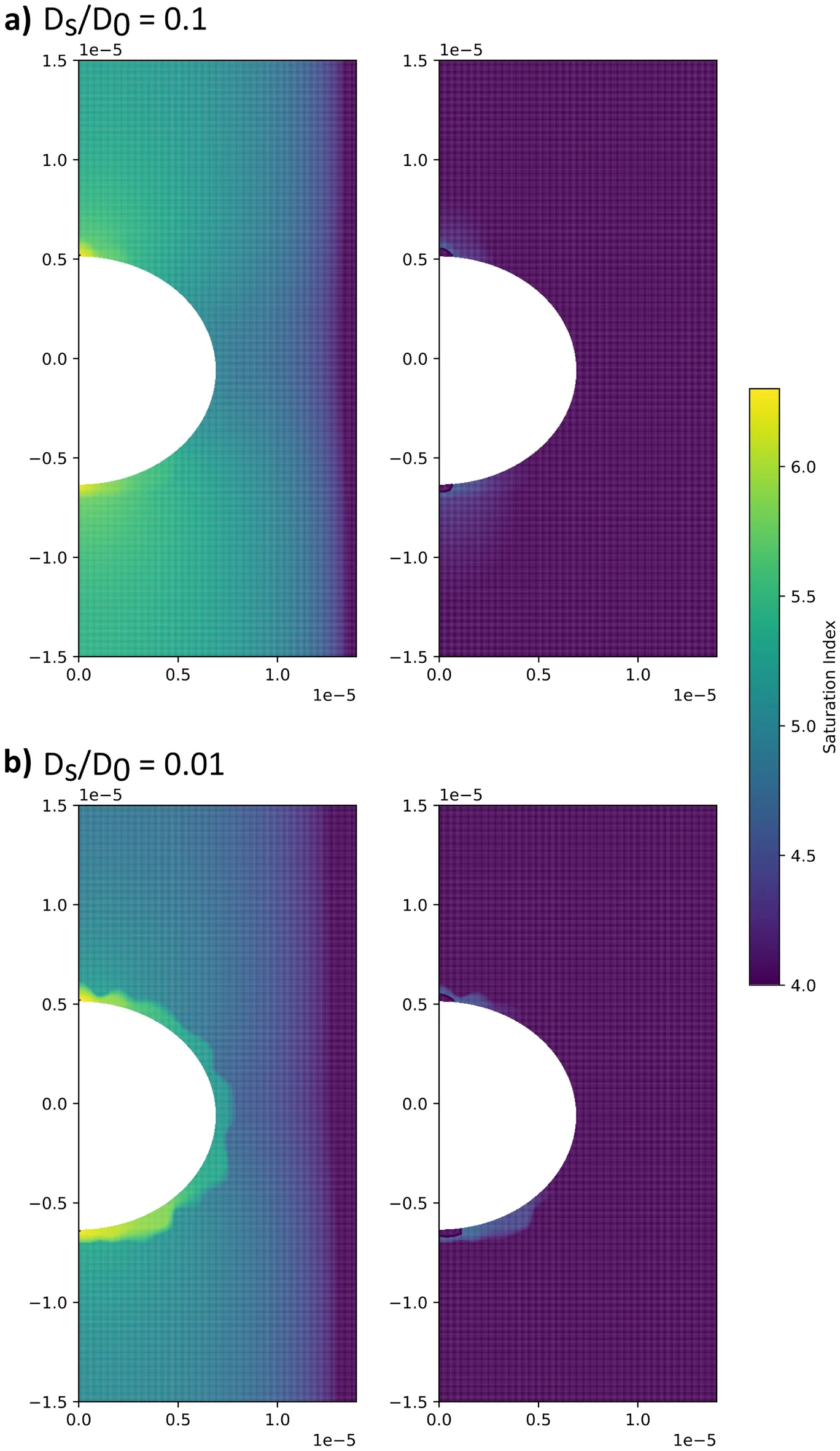
Modelled saturation index *SI* of Ba$^{2+}$ around a gas bubble. (**a**) *SI* at $D_{s}/ D_{0} = 0.1$ at initial stage of the model with high Ba$^{2+}$ concentration of 300 mM (left) and later stage of the model with Ba$^{2+}$ concentration of 10 mM (right). (**b**) *SI* at $D_{s}/ D_{0} = 0.01$ at initial stage of the model with high Ba$^{2+}$ concentration of 300 mM (left) and later stage of the model with Ba$^{2+}$ concentration of 10 mM (right).

A Lattice Boltzmann method simulating the diffusion of chemical species (in this case Ba$^{2+}$ ions) and evaluation of the corresponding saturation indices are performed directly on the structure in Fig. 1c. The model setup is described in subsection "Model definition for the investigation of saturation index and nucleation rate profiles" and uses pore solution compositions derived from experimental effluent analysis. The Ba$^{2+}$ concentration is initially 300 mM and decreases to 10 mM as the reaction progresses, whereas SO$_4^{2-}$ remains constant at 300 mM. The resulting *SI* profiles, computed for both high and low effective diffusivities and for corresponding high and low Ba$^{2+}$ concentrations, are shown in Fig. 2. The results reveal a strong dependence of the *SI* distribution on transport conditions, particularly diffusivity. At high source of Ba$^{2+}$, high diffusivity (D$_{s}/$D$_{0} = 0.1$, Fig. 2a) leads to a nearly uniform *SI* profile across the domain, whereas low diffusivity (D$_{s}/$D$_{0} = 0.01$, Fig. 2b) produces strongly localized supersaturation in the vicinity of the bubble. This contrast persists even with a lesser source of Ba$^{2+}$, although with a smaller magnitude. The localization of *SI* under low diffusivity reflects accumulation of Ba$^{2+}$ ions around the bubble, where transport from the source is limited. In this regime, a steep concentration gradient is developed, leading to a “confined region” of high supersaturation around the bubble. In contrast, when diffusivity is higher, Ba$^{2+}$ ions are uniformly distributed throughout the domain, eliminating localized supersaturation.

Taken together, these results indicate that the observed heterogeneity in the structure in Fig. 1c originates from diffusion-limited transport during the early-stage condition (300 mM Ba$^{2+}$). The evolving low-diffusivity region around the bubble acts as a restricted transport pathway that promotes local accumulation of Ba$^{2+}$ ions, generating persistent supersaturation gradients that become imprinted in the evolving structure. As the system evolves toward lesser source of Ba$^{2+}$ (late-stage condition, 10 mM Ba$^{2+}$), the overall driving force decreases, but the spatial organization established under diffusion-limited conditions is preserved. The final spatially heterogenous diffusivity state can therefore be interpreted as a residual feature of diffusion-controlled growth, governed by restricted transport and localized supply around the bubble.

### Nucleation mechanism in the experiment

**Fig. 3:**
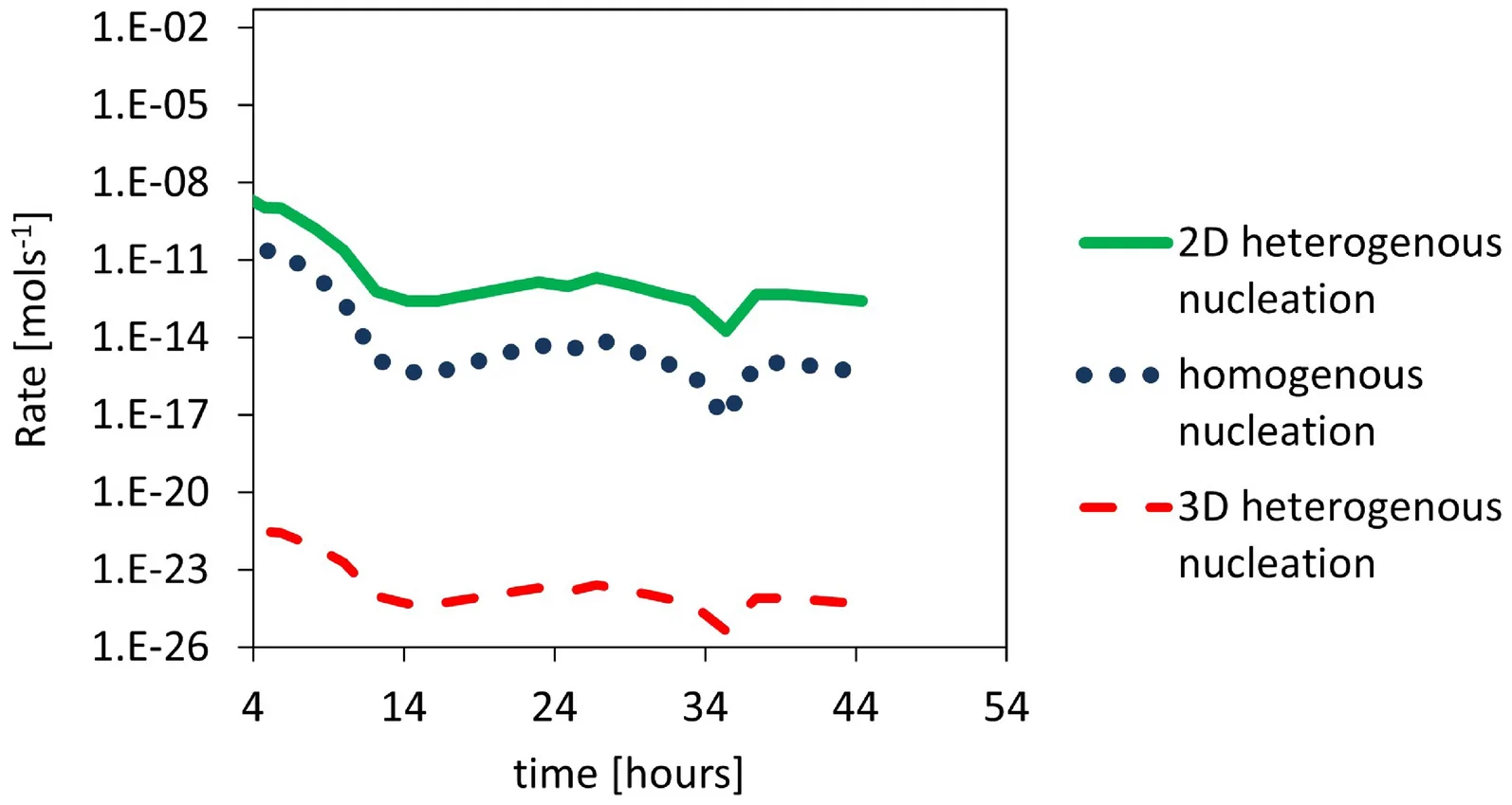
Nucleation mechanisms under our experimental conditions. Nucleation rate as a function of time, derived from the measured effluent composition. The 3D heterogenous nucleation computed here is for a contact angle of $\le 90^\circ$.

Using effluent concentration; rates for homogenous nucleation, 3D heterogenous nucleation, and 2D heterogenous nucleation were calculated as a function of time using classical nucleation theory (CNT), following the formulations of^24^ and its application to barite systems by^25^, with parameters constrained as in^26^. Homogenous nucleation occurs in the bulk solution within the pore space, whereas heterogenous nucleation takes place on surfaces. In the latter case, 3D heterogenous nucleation involves cap-shaped crystalline clusters forming at gas-liquid or solid-liquid interfaces, while 2D heterogenous nucleation describes the formation of disc-shaped crystalline clusters on pre-existing barite surfaces. The latter therefore requires an existing barite substrate and cannot initiate nucleation. Details of the governing equations are provided in Supplementary Note S5. The resulting nucleation rates are shown in Fig. 3 as a function of time (0–44 h). The calculated rates decrease with time, with 2D heterogenous nucleation yielding the highest rates, followed by homogenous nucleation, while 3D heterogenous nucleation remains negligible.

These results show that homogenous nucleation is possible, and barite precipitates were observed in the pore spaces through experimentation^8^. Homogenous nucleation exhibits a significantly lower free energy barrier than 3D heterogenous nucleation due to the geometric factor associated with the contact angle of barite nucleating on the surface of the gas bubble. Sensitivity analysis over contact angles ranging from 10–90$^\circ$ indicate that for contact angles $\le 90^\circ$, at which the results are shown in Fig. 3, the critical nucleus size approaches $n_c \le 1$, implying that the nucleation barrier is strongly reduced. Under these conditions, nucleation proceeds on short timescales compared to diffusion, resulting in the establishment of a mineralized interface.

The nucleation-rate analysis suggests that 2D heterogenous nucleation is favored once a barite substrate exists. Homogenous nucleation is predicted to dominate over 3D heterogenous nucleation, consistent with precipitation observed in the pore space (e.g., Fig. 1b). Nevertheless, the estimated induction time for 3D heterogenous nucleation at the bubble surface remains below 1 s under the assumed interfacial geometry (Supplementary Information Equation S7). Aqueous films adjacent to the bubble may additionally facilitate ion transport and create locally favorable conditions for nucleation^21^. Once the first barite crystallites form, subsequent growth may proceed predominantly through 2D nucleation on existing barite surfaces.

**Fig. 4:**
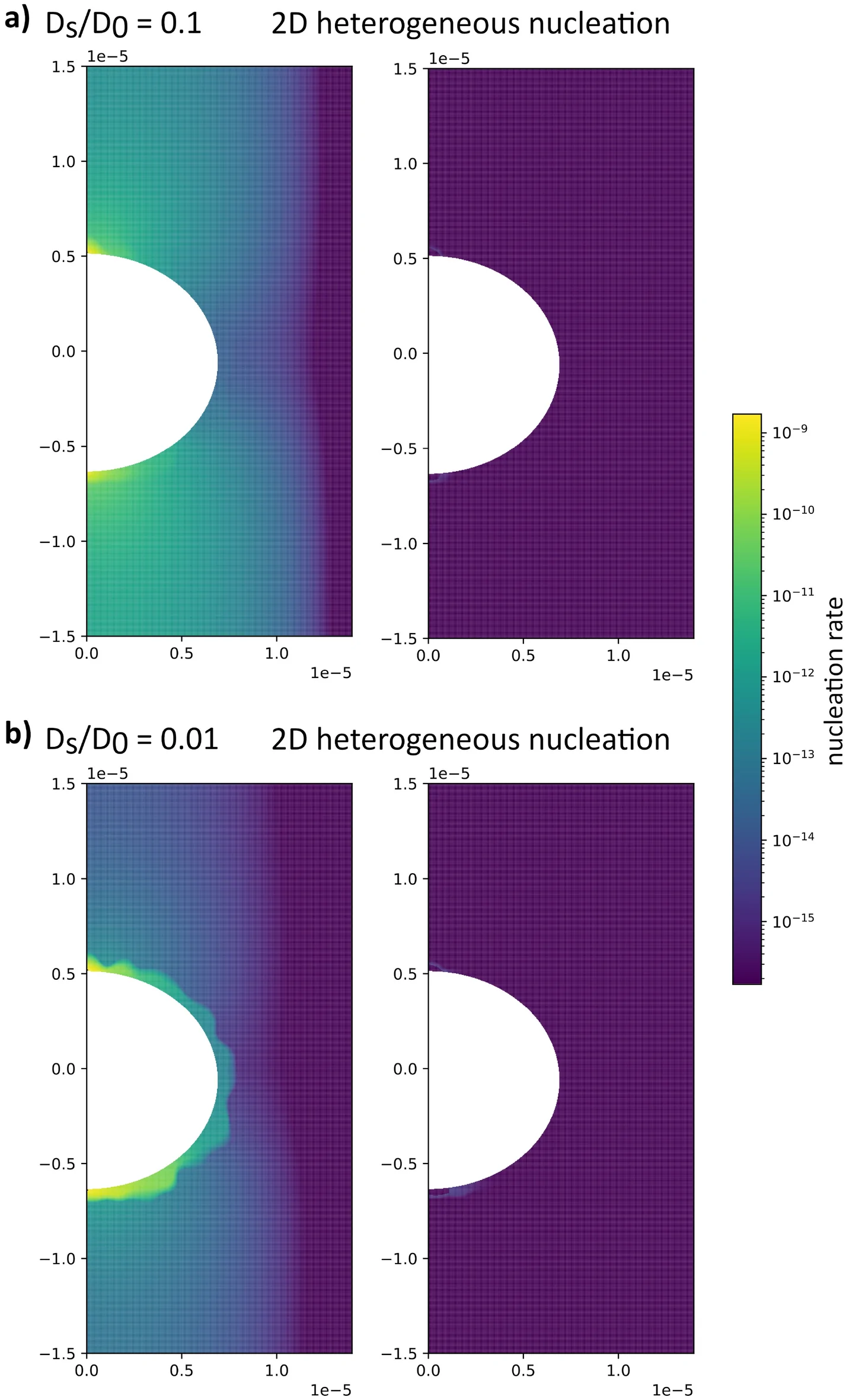
Modelled spatial 2D heterogenous nucleation. (**a**) 2D heterogenous nucleation at $D_{s}/ D_{0} = 0.1$ at initial stage of the model with high Ba$^{2+}$ concentration of 300 mM (left) and later stage of the model with Ba$^{2+}$ concentration of 10 mM (right). (**b**) 2D heterogenous nucleation at $D_{s}/ D_{0} = 0.01$ at initial stage of the model with high Ba$^{2+}$ concentration of 300 mM (left) and later stage of the model with Ba$^{2+}$ concentration of 10 mM (right).

In addition, the *SI* profiles shown in Fig. 2 were used to compute the corresponding nucleation rates [mol/s] around the bubble for both early-stage conditions (300 mM Ba$^{2+}$) and late-stage conditions (10 mM). Considering the calculated nucleation rates (Fig. 3), only the spatial distribution of 2D heterogenous nucleation around the bubble is presented in Fig. 4 and discussed in this section. The results for homogenous nucleation and 3D heterogenous nucleation are provided in Supplementary Figure S4. For high diffusivity within the precipitate, the associated 2D heterogenous nucleation rate is relatively uniform along the bubble interface (Fig. 4a), with a maximum near the edge closest to the Ba$^{2+}$ source. As the Ba$^{2+}$ ion concentration decreases, nucleation becomes increasingly localized, with significant activity restricted to regions proximal to the source. In contrast, lower diffusivity leads to a more heterogenous *SI* distribution, which produces a strongly heterogenous nucleation rate field (Fig. 4b). Under these conditions, barite accumulation increases while maintaining the initial spatial pattern, with preferential thickening observed near the Ba$^{2+}$ ion source.

### Geometrical analysis testing Mullins-Sekerka instability

To identify Mullins-Sekerka instabilities, one approach is to analyze the geometry of the secondary mineral phase. Following the analytical solution by Mullins and Sekerka^11,27^, the local radius of a spherical geometry subject to instability can be expressed as:

3 $$\begin{aligned} r = R + \delta \hspace{1ex} Y_{(l)}^{(m)}(\theta , \varphi ), \end{aligned}$$

with

4 $$\begin{aligned} Y_{(l)}^{(m)}(\theta , \varphi ) = \sqrt{\frac{2l+1}{4\pi } \frac{(l-m)!}{(l+m)!}} P_{(l)}^{(m)}(cos(\theta )) e^{im\varphi } \end{aligned}$$

where *R* denotes the average radius, *δ* is the perturbation amplitude, $Y_{(l)}^{(m)}(\theta , \varphi )$ represents the spherical harmonics defined in the spherical coordinates *θ* and *φ*, and $P_{(l)}^{(m)}$ is the associated Legendre polynomial. From Equation 3, under steady state approximation, absolute stability criterion indicating instabilities can be expressed as follows^11,27^:

5 $$\begin{aligned} \frac{R_{a}}{R} = 1 + \frac{1}{2}(l+1)(l+2)> 7, \end{aligned}$$

where $R_{a}$ denotes the absolute radius. Another criterion is the relative stability criterion, given by^11,27^:

6 $$\begin{aligned} \frac{R_{r}}{R} = \frac{l(l+1)^{2}-6}{2(l-2)}> 21, \end{aligned}$$

where $R_{r}$ denotes the relative radius.

For the Mullins-Sekerka analysis, a set of eight 2D SEM images of bubbles in the pore space was examined to ensure robust statistical characterization of interfacial morphologies. The mean wavelength for bubble perturbations is in the order of magnitude of $10^{-6}$ m for six bubbles and at $10^{-7}$ m for two. In contrast, the 3D FIB-SEM dataset is limited to three regions of interest and captures only partial bubble geometries (approximately half-bubbles). The geometrical analysis is therefore shifted to 2D in which the local radius of a circular geometry subject to instability can be expressed as:

7 $$\begin{aligned} r(\theta ) = R(\theta ) + \delta \hspace{1ex} cos(l \theta ) \end{aligned}$$

Instabilities can be identified by fitting the observed 2D morphology using Equation 7 to extract the predominant modes (utilizing Fourier transform). These modes are then fed into Equation 5 and 6 to evaluate the stability criteria and confirm the presence of instabilities. This approach is applicable because each observed 2D morphology represents a cross-section of the corresponding 3D morphology. Furthermore, additional instability evaluations using 2D stability criteria is presented in Supplementary Notes S6. The procedure for this evaluation is presented in Table 1.

**Table 1:**
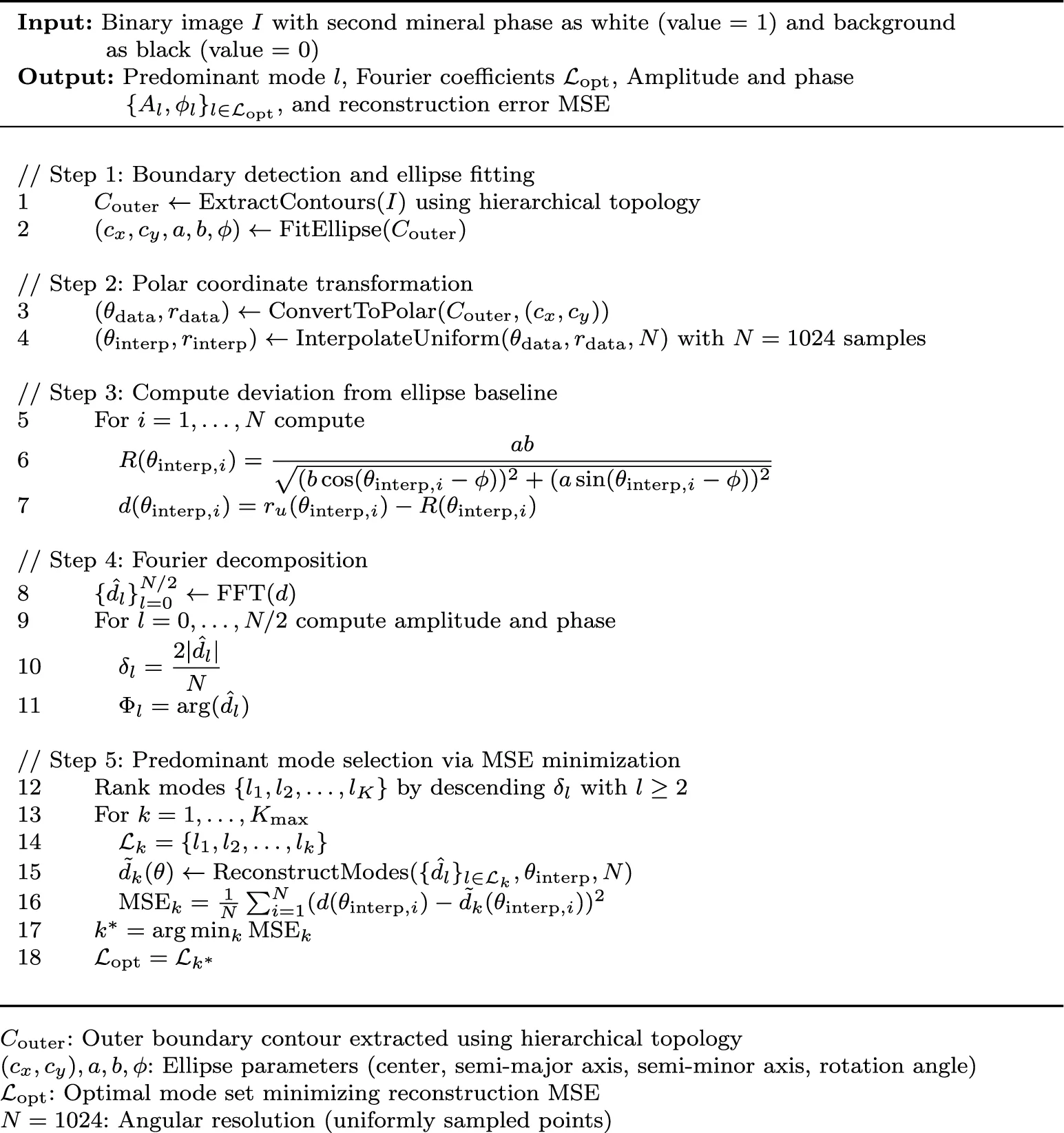
Procedure for evaluating morphological instabilities from a 2D SEM image.

**Fig. 5:**
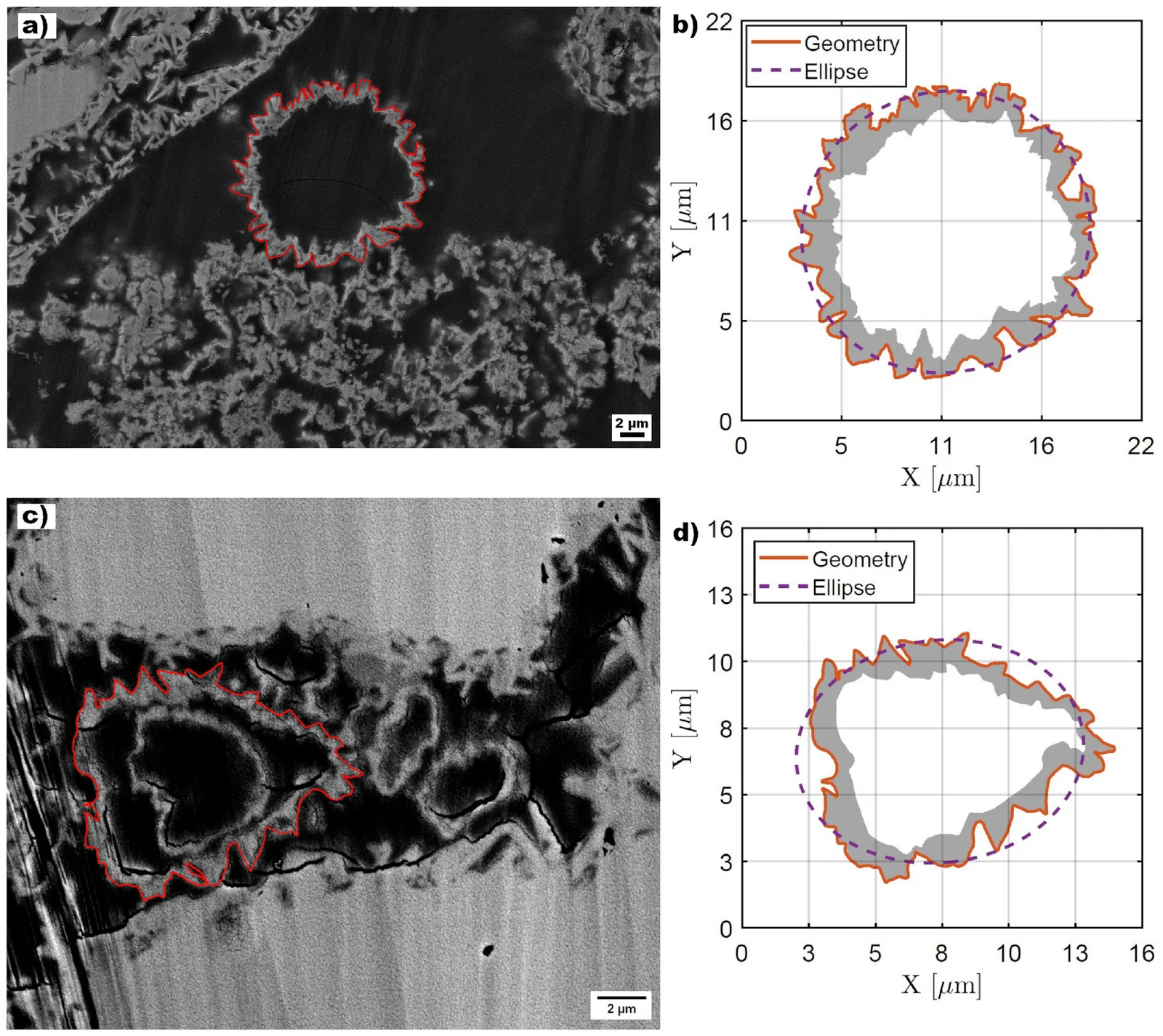
Evaluation of predominant modes from 2D SEM images. (**a**) SEM image of isolated bubble in the pore space. (**b**) Fourier decomposition of the isolated bubble with true outer boundary and fitted outer boundary marked to help identify dominant interface modes. (**c**) SEM image of deformed bubble in the pore space. (**d**) Fourier decomposition of the deformed bubble with true outer boundary and fitted outer boundary marked to help identify dominant interface modes.

Figure 5 presents the fitting results obtained using the procedure described in Table 1. The results in Fig. 5 show that the geometry of the secondary mineral phase in the SEM image in Fig. 5a can be adequately captured using Equation 7 (*MSE = 77.18* pixel$^{2}$) (Fig. 5b). The fitting yields 19 predominant modes, resulting in an absolute stability criterion of 191 and a relative stability criterion of 202.9. Because the geometry of the secondary mineral phase is consistent with the analytical formulation of Mullins-Sekerka instability described by Equation 7, the results indicate that the bubble morphology is consistent with Mullins-Sekerka-type behavior.

The observed geometry in the SEM image in Fig. 5c can not be replicated by the fitting results shown in Fig. 5d, not indicating the presence of Mullins-Sekerka-type behavior. The fitting yields only 3 predominant modes, resulting in an absolute stability criterion of 7 and a meaningless relative stability criterion. It also has a high *MSE* of 2450.12 pixel$^{2}$.

The remaining fitted geometries in Supplementary Figure S5 indicate the presence of bubble morphologies with Mullins-Sekerka-type behavior with a low absolute stability and relative stability criterion. This behavior is primarily attributed to deviations from the circular or elliptical geometry assumed in the analytical solution of Mullins-Sekerka. Wavelength perturbations alone do not show if Mullins-Sekerka-type behaviour can be observed morphological, justifying our analysis with the stability criterion. Refer to Supplementary Table S3 for reported modes, and 2D relative and absolute stability criterion.

## Discussion

Although the classical Mullins-Sekerka instability provides a robust theoretical framework for describing diffusion-controlled interfacial growth and has been validated under idealized conditions, our observations reveal systematic deviations from its predictions in pore-scale environments. These deviations do not invalidate the Mullins-Sekerka framework itself; rather, they highlight the emergence of additional controlling mechanisms that become dominant under confined and evolving conditions. In particular, the interplay between pore-scale confinement and capillarity, the time-dependent modification of effective diffusivity due to secondary mineral precipitation, and the role of heterogenous nucleation and crystallization kinetics collectively perturb the assumptions of a smooth interface and constant transport properties. The following sections examine how each of these factors progressively alter interfacial stability and growth dynamics, leading to the complex morphologies observed in our system.

As we have shown through the Fourier decomposition of the 2D SEM images (Section "Geometrical analysis testing Mullins-Sekerka instability") the surface morphology of the precipitate encrusted bubbles also deviate from Mullins-Sekerka instability. Our results show one bubble isolated in the pore space (Fig. 5a), exhibiting free spherical growth. However, various examples show that the bubbles have to grow in the confinement of pore space. Surrounding witherite grains or BaSO$_{4}$ precipitates influence the shape of the gas bubbles and consequently of secondary mineral precipitates. Studies show that there are multiple factors, such as phase connectivity, saturation state, and phase wettability, that control bubble generation, growth and transport. Nonetheless, capillarity is identified as the dominant factor that controls a bubble’s behaviour in pore space^28^. Capillary pressure ($P_{c}$) is derived from the Young-Laplace equation $P_{c} = \gamma k$ where *γ* is the interfacial tension and *k* is the surface curvature. This indicates that the capillary pressure decreases continuously with volume. However, capillary pressure is greatly influenced by geometry of the bubble dictated by the surrounding pore space as shown in experimental and theoretical studies^29–33^. Bubble deformation leads to heterogenous distribution of solutes, which leads to a heterogenous reaction front and therefore shows that the Mullins-Sekerka instability is not valid for such cases anymore.

Another source of deviation from the Mullins-Sekerka framework is the temporal evolution of effective diffusivity during mineral precipitation. The classical theory assumes constant and spatially uniform diffusivity throughout the development of the instability pattern^11,13,27^. This assumption is not satisfied in the present system. As precipitation proceeds, the formation of a porous barite layer progressively reduces the effective diffusivity at the reaction front. This reduction modifies the local mass flux, leading to the development of spatial gradients in supersaturation. As a result, the initially smooth diffusive field becomes increasingly heterogenous with time. This transition is directly reflected in the distribution of 2D heterogenous nucleation rates. At high effective diffusivity, nucleation is distributed relatively uniformly along the bubble interface. With decreasing diffusivity, nucleation becomes spatially confined, with activity concentrated in regions of sustained solute supply, particularly near the Ba$^{+2}$ source. The reaction front therefore evolves from a quasi-uniform to a localized regime. Consequently, the interface departs from the circular or elliptical morphologies predicted by the Mullins-Sekerka theory. Instead, growth becomes spatially differentiated, with preferential thickening in specific regions while the overall pattern is retained. The evolution of diffusivity thus introduces a dynamic coupling between transport and growth that is not captured by the classical framework.

Mineral precipitation at the gas-liquid interface is initiated by instantaneous precipitation or 3D heterogenous nucleation as the induction time remains below 1 s, leading to the early formation of a barite substrate. Once this substrate is established, crystallization proceeds on the solid surface rather than at the fluid interface, with surface growth kinetics controlling the local evolution of the interface. At the supersaturation conditions of the present study (*SI ≈ 5.2*−6.4), which exceed the critical threshold for 2D nucleation-dominated growth ($SI^* \approx 4.3$)^34^, crystallization is governed by rapid and spatially distributed 2D nucleation. In this regime, nuclei continuously form, spread, and coalesce across the surface, resulting in growth that is not confined to a limited number of defect-mediated sites but instead across the entire interface.

This behavior is consistent with the observed microstructure (Fig. 1d), where the mineral shell is composed of numerous small, platelet-like barite crystals. The resulting polycrystalline texture indicates that growth proceeds through repeated nucleation and lateral spreading of discrete crystallites, rather than through uniform layer advancement. Consequently, the interface deviates from the smooth and continuous boundary assumed in classical Mullins-Sekerka theory and instead exhibits spatially heterogenous growth conditions or surface roughening.

Pore-scale reactive transport simulations by Starchenko^20^ further support this interpretation, while studying barite growth from a sphere, showing that the inclusion of nucleation leads to spatially distributed precipitation patterns, with growth occurring at multiple dynamically evolving sites rather than along a uniform reaction front departing the system from Mullins-Sekerka instability. While nucleation has a limited impact on the overall precipitation rate, Starchenko shows that it strongly influences the spatial organization of the growing phase, causing a roughening of the interface.

In the present system, the combination of distributed nucleation, polycrystalline growth, and connected porosity leads to an interface that is both structurally and dynamically heterogenous. Under these conditions, interfacial evolution is governed by growth across a rough and transport-active surface, providing a mechanistic basis for the observed deviations from classical diffusion-controlled Mullins-Sekerka behavior.

## Methods

### Sample preparation

For this work, we collected a resin impregnated sample from the 3D reactive transport experiment from^8^. In this experiment we studied coupled mineral dissolution and precipitation processes with gas exsolution in a porous medium. The sample is cut in half lengthwise, then one half of the sample is cut through the middle of the layer of packed witherite grains (width = 0.5 cm). One Argon-Broad-Ion-Beam (BIB) cross-section is produced using a 90° slope-cutting technique in an JEOL SM-09010 device. The device is set at a 6 kV acceleration voltage for a duration of 8 hours. The BIB-milled area covers roughly 1 mm$^{2}$ of the reacted layer on the surface of the cross cut through the layer. The sample is used for SEM and FIB-SEM.

### SEM and FIB-SEM analysis

SEM analyses are performed at an accelerating voltage of 10 kV and a working distance of 8.5 mm. Back-scattered Electron (BSE) images are taken, giving an overview of the spatial distribution of the primary mineral phase (BaCO$_{3}$) and the secondary, precipitated mineral phase (BaSO$_{4}$). Based on SEM images three regions of interest (ROI) are defined for further analysis with FIB-SEM with special focus on an isolated gas bubble in the pore space covered with secondary mineral precipitates. Furthermore, eight BSE images of precipitate-covered gas bubbles are recorded to investigate their surface morphology. Gas bubbles of various sizes that are mostly isolated in the pore space are chosen for analysis.

FIB-SEM is performed using a FEI Helios NanoLab G3 (Thermo Fisher Scientific, Waltham, MA, USA). It is used to obtain tomography volumes using the sequential ion slice and electron imaging technique. BSE images are taken at an acceleration voltage of 5 kV and current of 0.1 nA. The region of interest (36.2 *μ*m x 24.2 *μ*m x 23.3 *μ*m) details isolated BaSO$_{4}$-precipitates around a CO$_{2}$ gas bubble. Refer to Supplementary Note S1 and Supplementary Figure S1 for details on a location showing the contact between primary and secondary mineral phases, and a location of a thick precipitation rim in the pore space.

FIB-SEM tomography investigations were performed within the EXCITE$^{2}$ project at the Electron Microscopy Centre of the University Utrecht.

### Image processing, quantification of porosity, and pore size distribution

Images are stacked, registered and cropped to size using Fiji (Image J 1.54f, Wayne Rasband and contributors, National Institutes of Health, Bethesda, MD, USA). The extensive image datasets collected from FIB-SEM are processed using the trainable Deep Learning model of the ZEISS arivis Cloud Software^35^ to make them binary. A porosity analysis of the binary images is performed using Fiji. Additionally, the FIB-SEM tomograph is reconstructed using the Dragonfly 3D World software (dragonfly, Comet Technologies Canada Inc). Figure 1a shows the 3D reconstructed FIB-SEM structure of the isolated gas bubble in the pore space. The view cross-cuts through the precipitates accessing the inside of the gas bubble. Refer to Supplementary Movie S1 available for a 360° spin of the 3D model of the gas bubble. We perform an analysis of the 3D-connected components of the pore space to visualize pore connectivity. Pore size analysis and distribution is performed with Microsoft Excel from pore volumes determined with Dragonfly. Pores are assumed to be spherical and the volume is used to back calculate pore diameter in the system. Refer to the Supplementary Note S2 for details of the image processing workflow.

### Bubble morphology

In order to perform morphology analysis of the surface of the precipitate covered gas bubbles, seven BSE images of variously sized bubbles in the pore space are processed with a paint-style application. We determine the inner radius of the bubbles covered in precipitates as the mean of radii in various directions of the different bubbles. This assumes that all bubbles are spheres and especially removes a bias from elliptical bubbles that grew non-spherical because of space availability around it. The wavelength (*λ*) of the wave-like pattern of the secondary mineral phase is determined from the processed image. Refer to Supplementary Note S2 for details of the image processing process and Supplementary Table S3 for radius and wavelength measurements.

### Evaluation of effective diffusivity

We employed Lattice Boltzmann simulations to evaluate the effective diffusivity of the stacked images using a D3Q7 scheme. The simulation solves for distribution functions *f* as follows^21,36^:

8 $$\begin{aligned} f_{k}(\textbf{r} + c_{D} \textbf{e}_{k} \delta _{x}, t+\delta _{t,D}) - f_{k}(\textbf{r}, t) = - \frac{1}{\tau _{D}} \left[ f_{k}(\textbf{r}, t) - f^{eq}_{k}(\textbf{r}, t) \right] + S, \end{aligned}$$

where $\textbf{r}$ [m] denotes the position vector, $\textbf{e}$ is the unit discrete velocities, $\delta _{x}$ [m] is the grid size, $\delta _{t,D}$ [s] is the time step, $c_D=\delta _{x}/\delta _{t,D}$ [m/s] is the lattice speed, and *S* is the source term (in our case *S = 0*). $\tau _{D}$ [-] is the dimensionless relaxation time given by $\tau _{D}=4D/c_D\delta _{x}+0.5$. The subscript *k = 0, 1, 2,..., 6* corresponds to the directions in the *Q = 7* scheme (D3Q7). The superscript *eq* denotes equilibrium distribution functions.

The equilibrium distribution functions are defined as follows^21,36^:

9 $$\begin{aligned} f^{eq}_{k}(\textbf{r}, t) = \omega _{k} C(\textbf{r}, t_{0}), \end{aligned}$$

with

10 $$\begin{aligned} \omega _{k} = {\left\{ \begin{array}{ll} \frac{1}{4} & k = 0\\ \frac{1}{8} & k = 1 - 6 \end{array}\right. }. \end{aligned}$$

Here, $C(\textbf{r}, t_{0})$ [mol/m$^{3}$] denotes the initial spatio-temporal tracer concentration. The local tracer concentration at any position and time $C(\textbf{r}, t)$ is calculated by $C(\textbf{r}, t) = \sum _{k} f_{k}$. The local flux along *x* direction $\textbf{J}_{x}$ is calculated locally through $f_{k}$:

11 $$\begin{aligned} \textbf{J}_{x}= \frac{\tau _{D} - 0.5}{\tau _{D}} \frac{\delta _x}{\delta _{t,D}} \sum _{k} \left( \textbf{e}_k \cdot \textbf{e}_x \right) f_{k}(\textbf{r}, t). \end{aligned}$$

To evaluate the effective diffusivity, a constant concentration boundary condition is imposed along the *x* direction, with inlet concentration *C(x = 0, t) = 1.0* [mol/m$^{3}$] and outlet concentration $C(x = L_{x}, t) = 0.0$ [mol/m$^{3}$]. When the simulation reaches stationary, the effective diffusivity is determined through the following equation:

12 $$\begin{aligned} D_e=\frac{L_{x}}{C(x = 0, t \rightarrow \infty )-C(x = L_{x}, t \rightarrow \infty )} \frac{\sum \textbf{J}_x}{S}, \end{aligned}$$

where $L_{x}$ is the length of the sample.

### Model definition for the investigation of saturation index and nucleation rate profiles

**Fig. 6:**
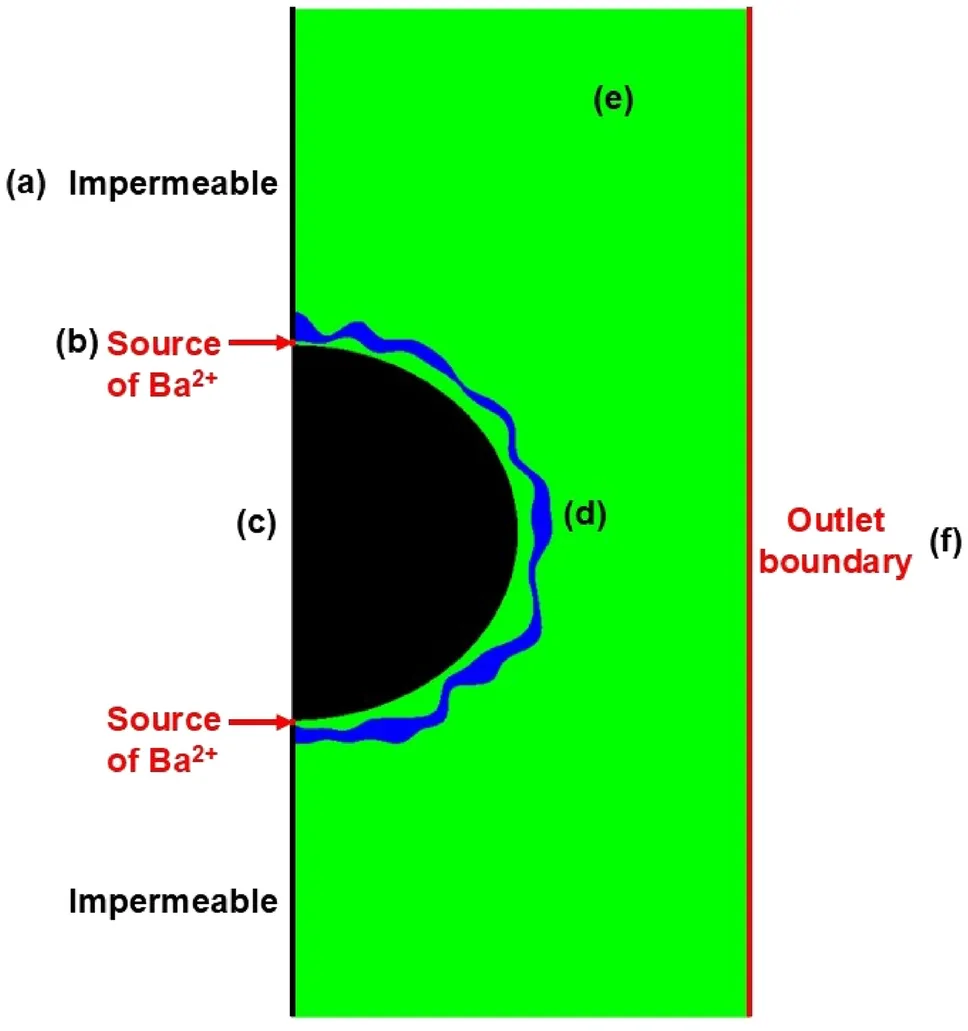
Computational domain for the evaluation of saturation indeces and nucleation rates. (**a**) Impermeable barrier, (**b**) source of Ba$^{2+}$ from dissolution, (**c**) impermeable gas bubble, (**d**) precipitate with reduced diffusivity, (**e**) water-filled pores, (**f**) outlet boundary for Ba$^{2+}$.

To investigate the evolving diffusivity in subsections "Consequences of evolving diffusivity on the saturation index at the gas-liquid interface" and "Nucleation mechanism in the experiment", we employ a coupled Lattice Boltzmann simulation and chemical equilibrium calculation. We used our open-source Fortran-based Lattice Boltzmann solver to simulate single-component (Ba$^{2+}$) diffusion, and the open-source Python-based Reaktoro framework^37^ as the chemical equilibrium solver for computing *SI*. The governing equations solved by the Lattice Boltzmann method are given in Equations 8 to 10. The model setup is as follows:

1. The geometry from Fig. 1c is used directly and coarsened to reduce computational cost while preserving the essential physical features, shown in Fig. 6. Impermeable barriers are assigned above and below the precipitate as diffusion of Ba$^{2+}$ ions can only occur around the gas bubble (see Fig. 6a).
2. Ba$^{2+}$ ion is the only diffusing chemical species considered, as the gas bubble forms through dissolution of witherite, which releases Ba$^{2+}$ ions. Due to continuous Na$_{2}$SO$^{4}$ injection, the concentration of SO$_{4}^{2-}$ is assumed constant at 300 mM^8^.
3. As the gas bubble forms from witherite dissolution and does not permit Ba$^{2+}$ transport, a Dirichlet boundary condition is imposed at the inlet (left boundary in Fig. 6b) between the gas bubble and the precipitate. Two inlet concentrations (300 mM, reflecting early-stage condition and 10 mM, reflecting late-stage condition) are prescribed based on effluent history^8^.
4. The diffusivity of the precipitate phase (see Fig. 6d) is varied (D$_{s}/$D$_{0} = 0.1$ and 0.01).
5. A zero-concentration Dirichlet boundary condition is imposed at the outlet boundary (right boundary in Fig. 6f). We ensured that the diffusion front does not reach the outlet boundary.
6. The Lattice Boltzmann simulation was run until stationary, then saturation index profile is calculated using this stationary concentration.

## Supplementary Information

Supplementary Information 1.

Supplementary Information 2.

## Data Availability

The data used for the presented experiments in the study are available at Jülich DATA via doi.org/10.26165/JUELICH-DATA/WRKGA0 (Wegner et al., 2026) with Creative Commons CC-BY 4.0 - ”Attribution” with no further registration required.

## Code availability

The codes for performing the Lattice Boltzmann simulation are available in the Github repository https://github.com/FZJ-RT/lbm.git.
